# Survey of rodent-borne pathogens in Singapore reveals the circulation of *Leptospira* spp., Seoul hantavirus, and *Rickettsia typhi*

**DOI:** 10.1038/s41598-021-03954-w

**Published:** 2022-02-17

**Authors:** Jane Griffiths, Hui Ling Yeo, Grace Yap, Diyar Mailepessov, Patrik Johansson, Hwee Teng Low, Chern-Chiang Siew, Patrick Lam, Lee Ching Ng

**Affiliations:** 1 Environmental Health Institute, National Environment Agency, Singapore, Singapore; 2 Defence Medical and Environmental Research Institute, DSO National Laboratories, Singapore, Singapore; 3 SAF Biodefence Centre, Force Medical Protection Command, HQ Medical Corps, Singapore Armed Forces, Singapore, Singapore

**Keywords:** Molecular biology, Zoology, Diseases, Health care, Risk factors

## Abstract

Rodents living alongside humans increases the probability of encounter and also the transmission of rodent-borne diseases. Singapore’s cosmopolitan urban landscape provides a perfect setting to study the prevalence of four rodent-borne pathogens: Seoul hantavirus (SEOV), *Leptospira* species, *Rickettsia typhi* and *Yersinia pestis*, and identify the potential risk factors which may influence rodent density and transmission of rodent-borne diseases. A total of 1143 rodents were trapped from 10 unique landscape structures throughout Singapore. Real-time quantitative Polymerase Chain Reactions were used to detect pathogenic and intermediate *Leptospira* spp. and *Yersinia pestis*, whereas the seroprevalence of SEOV and *R. typhi* were analysed by Enzyme-Linked Immunosorbent Assay and Immunofluorescence Assay respectively. Multivariable logistic regression analysis was used to evaluate the association between prevalence of infection in rodent reservoirs and risk factors. Most of the rodents were caught in public residential developments (62.2%). Among the tested rodents, 42.4% were infected with *Leptospira* spp., while 35.5% and 32.2% were seropositive for SEOV and *R. typhi* respectively, whereas *Yersinia pestis* was not detected. Furthermore, risk factors including habitat, species, gender, and weight of rodents, influenced prevalence of infection to a varying extent. This study highlights the presence of *Leptospira* spp., SEOV and *R. typhi* in Singapore’s rodent population, suggesting the need for effective rodent management and sanitation strategies to prevent further circulation and transmission to humans.

## Introduction

Rodents are well-known hosts and vectors for zoonotic pathogens^1^ and can spread diseases through fleas, excreta and their bites^2–4^. *Rattus norvegicus* (Norway rats or sewer rats) and *Rattus rattus* (black or roof rats) have lived in close association with humans for thousands of years^5,6^. Both *R. norvegicus* and *R. rattus* are thought to have originated in Asia^7,8^ but today, both species can be found worldwide^9,10^, being introduced from continent to continent along with human migration and trade^6,8^. Rodents are ubiquitous and highly adapted to modified and fragmented environments^11,12^ and thus urbanization has facilitated a close proximity between rodents and humans, increasing the risk of human exposure to the pathogens that rodents carry^13^.

Singapore is a densely populated^14^, modern city state, with residential developments intermingled with food establishments, nature parks, commercial sites and industrial areas. Building construction and shipbuilding activities are rampant. The urban landscape provides opportunities for harbouring rodents. Commonly found species, *Rattus norvegicus* can be found exploiting infrastructures such as sewers systems^15^, false floors and burrowing under buildings and concrete^16,17^ while *Rattus rattus*, known to be agile climbers^18^, can be found indoors^19^ taking refuge in false ceilings and wall cavities^20^. Both species are known to be carriers of several rodent-borne zoonotic pathogens including *Leptospira*, hantaviruses, rickettsiae and *Yersinia pestis* and these pathogens are known to cause disease in humans in Southeast Asia as well as globally^21–25^.

Leptospirosis is a common zoonotic disease worldwide^26^ caused by pathogenic species of *Leptospira*, and both wild and urban rodents are considered to be important reservoirs^27–29^. In recent years, there has been an increasing number of studies reporting the isolation of intermediate *Leptospira* spp., other than pathogenic *Leptospira* spp., from patients with symptoms consistent with leptospirosis^30,31^. Among the leptospirosis associated hospitalizations, patients can present a variety of flu-like symptoms such as fever, headache and chills to more severe symptoms, including kidney or liver failure and meningitis, with a 4–22% case fatality rate in cases with severe clinical illness^32–34^. An evaluation of the leptospirosis data collected between 2012–2015 by the Ministry of Health, Singapore, reported that there were 165 cases, of which 60% were locally acquired^35^. The occupational risk of leptospirosis infection was previously reported by Chan et al. in abattoir workers, cleaners and sewer workers^36,37^.

Hantaviruses have been estimated to have caused 60,000–150,000 cases of human haemorrhagic fever with renal syndrome (HFRS) worldwide, annually^38^. There are at least 22 hantaviruses known to be pathogenic to humans^22^ and various rodent species can act as a reservoir^39,40^, including common urban rodents found in Singapore^25^. Seoul hantavirus (SEOV) was reported to be carried by *Rattus norvegicus* and *Rattus rattus*, and is believed to cause milder disease than other hantaviruses in human host, leading to diagnostic challenge^41,42^. Hantavirus HFRS has previously, but rarely, been reported in Singapore^43,44^. Serological studies done in the late 1980s on wild rats as well as patients suspected of having dengue, leptospirosis or hepatitis revealed 26% (37/142) of rodents and 3.8% (32/836) of human patients were seropositive to hantavirus using immunofluorescence assay (IFA)^25^. Occupational risk groups for hantaviruses include forestry workers, construction workers^45^, and farmers^39^.

*Rickettsia typhi*, the causative agent of murine typhus, is known to cause a relatively mild febrile disease in humans but complications can arise resulting in death^46,47^. Infected rodents serve as an amplifying host for the bacteria, and the Oriental rat flea (*Xenopsylla cheopis*) acts as the main vector responsible for the transmission to humans^48–50^. In Singapore, murine typhus cases ranged annually between 19–128 human cases between the years 1999–2003^51^. Both occupational and domestic exposure to rodents has been highlighted in local case studies^52^, and construction workers and their living quarters are also commonly associated with this disease^52,53^.

Plague, a disease caused by an infection with the bacteria, *Yersinia pestis*, is a severe disease with a case fatality rate between 30–100% if left untreated^54^. Outbreaks of plague still occur in parts of the world, particularly Madagascar^55^, but there have been no reports in Singapore. In endemic areas, plague can be found circulating in the rodent population, while infected fleas serving as the main vector is involved in the transmission of *Y. pestis* to new rodent host or jumping to human host^56^.

While plague can cause severe life-threatening diseases, infections caused by HFRS-related hantaviruses, leptospirosis and murine typhus infections can range from mild and self-limiting diseases to acute, life-threatening diseases. Furthermore, these diseases often exhibit non-specific symptoms (including high fever, myalgia, headache, thrombocytopenia, rash, abdominal pain, nausea, malaise, anorexia and jaundice), making it difficult to differentiate between these and many other diseases^26,57–61^.

A study was conducted to assess the prevalence of these four rodent-borne pathogens in Singapore’s rodent population, and to analyse the characteristics of rodents, including habitat, weight, gender and rodent species, and their association with the prevalence of infection with the pathogens described above. This information may highlight areas of concern for public health in occupational and domestic situations, and for tailoring sanitation and rodent intervention strategies.

## Methods

### Ethics statement

All protocols were carried out according to relevant ARRIVE (Animal Research: Reporting of In Vivo Experiments) guidelines and regulations. Animal work was approved by Defence Science Organisation (DSO) National Laboratories Institutional Animal Care and Use Committee (DSO/IACUC/05/13) and performed in compliance with the National Advisory Committee for Laboratory Animal Research (NACLAR) Guidelines, Singapore.

### Study area and rodent sampling

Singapore is a densely populated island city-state in Southeast Asia with 7810 people per square kilometres. It has a tropical rainforest climate with warm temperatures, high humidity, and abundant rainfall, with little variation throughout the year. Rodent sampling took place between January 2006 and March 2008.

Live rodents were either obtained via opportunistic sampling conducted by pest control professionals registered with the Singapore Pest Management Association or from the research teams own trapping throughout Singapore. Trap locations were selected based on rodent related reports provided by the town councils, who are responsible for the management and maintenance of the common property.

Metal single-capture live traps were baited with barbequed dried cuttlefish and apple and placed at each trap location where there were reported signs of rodent activity. Traps were left overnight and checked the following morning. Rodents were brought back to the laboratory to be sampled within the same day. Traps were deployed for 1–2 weeks at each trap location due to low trap rate caused by either avoidance of the traps or reduction in rodent population as a result of active trapping.

Rodents were euthanized with carbon dioxide. Blood samples were collected by cardiac puncture and the resulting sera were kept at  – 20  °C until serological analysis. Location of capture, species (according to external morphology), body length, weight and sex of each captured animal were recorded. Spleen and kidney tissues were collected using aseptic techniques and kept frozen at –80 °C until DNA extraction.

### Sampling locations

Rodents’ urban habitats were categorized according to the features of the urban built environment such as landscape structure and variations in land use. Rodents were sampled from 10 unique landscape structures, spanning over 14 town councils of the total 16 town councils in Singapore. The following classifications were used:

*Army Camps-* a place for military training, where three to four-storey low rise buildings with living quarters, are surrounded by large green areas and vegetation. Living quarters are usually without air-conditioning, and are equipped with basic amenities such as canteen, communal dining, laundry, sanitary and bathing facilities.

*Commercial sites-* shophouses are generally two to three-storeys in height. The ground floor has been designed to accommodate commercial enterprises (non-food businesses), while the upper floors are usually intended for residential use.

*Construction sites-* premises with ongoing construction activities, construction temporary quarters (CTQs) with communal facilities (e.g. cooking, laundry, sanitary and bathing facilities) may be present to house workers of the site. Maximum occupancy of CTQ ranges from 6 to 300 workers, depending on the nature of the projects.

*Food establishments-* places selling fresh ingredients or prepared foods including restaurants, wet markets and hawker/food centres. Wet markets and food centres are usually one to two-storeys open-air complexes, with some housing as many as 200 vendors.

*Industrial areas-* premises include factories and warehouses for manufacturing purposes, usually with a nuisance buffer from residential areas. Staff canteens are available within industrial developments to primarily serve workers during breaks.

*Parks-* large green areas with vegetation. Bins are located at designated points around the park, with a scheduled waste collection service.

*Residential- private-* include landed and cluster houses, usually one or two-storeys in height, and condominiums. Usually surrounded by green areas with vegetation.

*Residential- public-* high-rise and high-density public housing managed by Housing Development Board (HDB). These buildings are generally ten to forty-storeys in height, with a centralised refuse chute leading to a large collection bin on the ground floor of each building. Manholes on the ground floor serve as access points for maintenance of building’s sewer pipes.

*Schools-* institutional buildings, usually four-storeys in height, surrounded by green areas such as gardens and grass fields with running tracks. Canteens have around ten food stalls, where food is prepared and sold during lunch breaks.

*Shipyards-* a place for shipbuilding and ship repair activities. Sites are near the sea to allow easy access for the ships.

### Rodent speciation

#### Species-specific Restriction Fragment Length Polymorphism (RFLP) analysis

DNA was extracted from 25 mg of kidneys using the DNeasy tissue kit (QIAGEN, Germany) according to the manufacturer’s instructions. Extracted DNAs were subjected to Polymerase Chain Reaction (PCR) using primers EGL3 and EGL4 to amplify the entire D-loop containing region of the mitochondrial DNA^62,63^ for rodent species identification. Each PCR reaction contained 5.0 µl of a prepared primer mix (with final concentration of each primer being 1 mM), 1.0 µl DNA template, 1.0 µl MgCl_2_ (50 mM), 0.25 µl dNTPs (100 mM), 0.1 µl *Taq* DNA polymerase (5 U/µl; Thermo Fisher Scientific, USA), 2.5 µl 10 × *Taq* Buffer and water to a final volume of 25 µl. The PCR reactions were performed using Biometra T-gradient (Biometra, USA) with an initial holding temperature of 94 °C for 60 s, followed by 35 cycles of 94 °C for 45 s, 60 °C for 60 s and 72 °C for 60 s, and 1 cycle of 5 min at 72 °C.

Half of the resulting PCR-reaction was transferred to a new tube for digestion with the DNA restriction enzymes *Hae*III (10 U) and *Dde*I (1.25 U) (Vivantis, Malaysia) and was incubated at 37 °C overnight. 10 µl of both digested and non-digested PCR-reaction were analysed on a 1.5% agarose gel stained with ethidium bromide and visualised under UV. The expected fragment sizes for the various *Rattus* spp. were as follows: *Rattus norvegicus* (376 base pairs (bp), 443 bp), *Rattus rattus* (183 bp, 301 bp, 391 bp) and *Rattus exulans* (376 bp, 575 bp)^62^.

#### PCR of animal cytochrome *b* gene for *Rattus* spp. identification

Samples identified as non- *Rattus norvegicus* using the RFLP-method were subjected to further PCR and sequencing of the mitochondrial cytochrome *b* (*cytb*) gene to discriminate between closely related *Rattus* species. The PCR and sequencing of *cytb* gene were performed using primers mcytbHb (5’-GAATGGGAGAATGAAGTGGAATGCG-3’) and mcytb1 (5’-CCATCGTTGTAATTCAACTATAG-3’)^8^. Each PCR reaction contained 1.5 µl of a prepared primer mix (with final concentrations of each primer being 2.5 µM), 1.5 µl extracted DNA from kidneys, 15 µl 2 × SYBR green master mix (Roche, Switzerland) and water to a final volume of 30 µl. The PCR reactions were performed and analysed using LightCycler 2.0 (Roche, Switzerland), with an initial holding temperature of 95 °C for 1 min, followed by 30 cycles of 95 °C for 30 s, 55 °C for 45 s and 72 °C for 1 min, and 1 cycle of 10 min at 72 °C. Obtained PCR amplicons were purified using QIAquick PCR Purification Kit (QIAGEN, Germany) and sequenced using both the PCR amplification primers. The resulting DNA sequencing chromatogram were assembled using SeqMan Pro (DNASTAR Lasergene, USA). The sequences derived was aligned against the *cytb* gene sequence entries in GenBank using the online BLAST search engine of the National Center for Biotechnology Information (NCBI) to detect sequences with high similarity for species identification.

### Pathogenic and intermediate *Leptospira* spp. DNA detection using specific PCR

A real-time quantitative PCR previously described by Smythe LD et al.^64^ was used for the detection of pathogenic and intermediate *Leptospira* spp.. Briefly, forward primers LeptoF (5’ – CCCGCGTCCGATTAG – 3’) and reverse LeptoR (5’ – TCCATTGTGGCCGRACAC – 3’) with probe (5’ FAM -CTCACCAAGGCGACGATCGGTAGC – 3’ TAMRA) were used to target a partial sequence of the *rrs* (16S) gene, that is present in both pathogenic and intermediate *Leptospira* spp.^65^. Real-time PCR assay was adapted to suit the LightCycler 2.0 system (Roche, Switzerland). Each PCR reaction consisted of 0.6 µl of a prepared primers and probe mix (with final concentrations of each primer/probe being 0.2 µM), 5.0 µl extracted DNA from kidneys, 2.0 µl 5 × LightCycler Multiplex DNA master mix (Roche, Switzerland) and water to a final volume of 10 µl. The PCR reactions were performed and analysed using LightCycler 2.0 (Roche, Switzerland), with an initial holding temperature of 95 °C for 2 min, followed by 55 cycles of 94 °C for 10 s and 60 °C for 60 s, and 1 cycle of 20 s at 40 °C. A negative result was assigned where no amplification occurred when threshold cycle (Ct) value was greater than 40 cycles.

### Serology assay for Seoul hantavirus

An Enzyme-Linked Immunosorbent Assay (ELISA) was performed on rodent sera as previously described^66^, for the detection of hantavirus-specific antibodies using a recombinant peptide antigen consisting of the 120 N-terminal amino acids of Seoul hantavirus nucleocapsid (N) produced in *E. coli* (obtained from Dr Göran Bucht)^67^. Briefly, high binding ELISA 96-well plates (Nunc, Thermo Fisher Scientific, USA) were coated with 50 µl of 1 µg/ml truncated Seoul hantavirus nucleocapsid protein in coating buffer (0.015 M Na_2_CO_3_, 0.034 M NaHCO_3_, pH9.6), for 2 h at 37  °C or overnight at 4  °C. The plates were blocked with 350 µl of 5% skim milk in Phosphate Buffered Saline (PBS), pH 7.4, for 1 h at 37  °C. Rodent sera were diluted 1:200 in PBS with 3% skim milk before 50 µl of each sample was added into each well and incubated for 2 h at 37  °C. The plates were washed twice with PBST (PBS supplemented with 0.05% Tween-20; Thermo Fisher Scientific, USA) and once with PBS. 50 µl of 1:10,000 dilution of HRP-labelled goat anti-rat (Pierce Biotechnology, Thermo Fisher Scientific, USA ) was added into each well and incubated 1 h at 37  °C. Plates were washed as previously described and 100 µl of TMB One Solution (Promega, USA) was added and the reaction was stopped after 15 min using 100 µl 2 M H_2_SO_4_. The plates were read at 450 nm in an ELISA plate reader.

### Immunofluorescence assay for detecting *Rickettsia typhi* IgG antibodies

Indirect Immunofluorescence Assay (IFA) is the golden standard for serodiagnosis of murine typhus. Preparation of the IFA slides and the IFA tests were done according to Bozeman FM et al.^68^. *Rickettsia typhi* (Wilmington strain) was grown in the Biosafety Level 3 laboratory. Briefly, 2 µl of substrate antigens (105 PFU/ml) were spotted on each well. The slides were air-dried and fixed with 80% acetone and frozen until usage. Rodent sera were diluted at least 1:64 in 2% Casein buffer (Sigma-Aldrich, USA), and 2 µl of each sample were spotted onto the slides. *R. typhi* positive controls and negative controls were included on each slide. Slides were incubated in a humidifier at 37  °C for 30 min. Goat anti-rat IgG conjugated to FITC (Chemicon, Merck, USA) was used as a detection antibody. The slides were further incubated at 37  °C for 30 min before being read using a fluorescence microscope. Fluorescence detected in rodent sera at 1:128 dilution was considered positive for *R. typhi* IgG antibodies.

### *Yersinia pestis* DNA detection using specific PCR

DNA was extracted from 10 mg of spleen using the DNeasy tissue kit (QIAGEN, Germany) according to manufacturer’s instructions. Extracted DNAs were subjected to real-time quantitative PCR for the detection of *Yersinia pestis*^69^. The real-time PCR assay was adapted to suit the LightCycler 2.0 system (Roche, Switzerland). Briefly, forward primers YP3a (5’ – TGTAGCCGCTAAGCACTACCATCC – 3’) and reverse YP3a (5’ – GGCAACAGCTCAACACCTTTGG – 3’) with probe (5’ FAM – TCAAGGTTATTGACGGTATCGAGTAGGGT – 3’ TAMRA) were used. Each PCR reaction consisted of 0.3 µl of a prepared primer mix (with final concentrations of each primer being 0.3 µM), 0.2 µl probe (final concentration of 0.2 µM), and 1.0 µl of extracted DNA, 2.0 µl 5 × LightCycler Multiplex DNA master mix (Roche, Switzerland) and water to a final volume of 10 µl. The PCR reactions were performed and analysed using LightCycler 2.0 (Roche, Switzerland), with an initial holding temperature of 95 °C for 1 min, followed by 55 cycles of 94 °C for 5 s, 60 °C for 5 s and 72 °C for 1 min, and 1 cycle of 30 s at 40 °C. A negative result was assigned where no amplification occurred when threshold cycle (Ct) value was greater than 40 cycles.

### Statistical analysis

To investigate the relationship between positive binary outcomes for *Leptospira* spp. PCR, Seoul hantavirus ELISA and *Rickettsia typhi* IFA and characteristics of rodents, three separate multivariable logistic regression models were built. The following variables were considered: the landscape structures where the rodents were trapped, species of the rodent, weight, and sex. As described above, there were 10 landscape structures: residential- public, residential- private, food establishments, construction sites, industrial areas, commercial sites, schools, army camps, parks and shipyards. Weight was coded as continuous variable, and the rest of the independent variables as categorical.

For each outcome, bivariable logistic regression analysis was first conducted to ascertain the significant association of the variable and the outcome. The bivariate analysis for the weight of rodents was restricted to species as *R. rattus* and *R. norvegicus* differ in size^70^. Subsequently, to avoid confounding between the explanatory variables and the outcome, multivariable logistic regression models for each outcome were built. Statistically significant variables with lowest *p*-values were added into the multivariable regression model first. The model was re-fitted after addition of each new variable. The retention of the variables in the model was justified if the variables remained statistically significant and the Akaike's Information Criterion value for the new model was lower compared to the model without the new variable^71^. Statistically significant cut off was set at 0.05. Since the weight differs between *R. rattus* and *R. norvegicus*, the interaction between weight and species was also investigated in the multivariable logistic regression model. The collinearity between the independent variables in all three regression models was assessed with the Generalised Variance Inflation Factor (GVIF^1/[2*df]^)^72^. Variables with values of GVIF exceeding threshold of 2 were removed from the final model^73^.

The analysis was conducted using R software version 3.6.3. Only complete observations were included in the final multivariable logistic regression models for each pathogen outcome. Multivariable logistic regression models with positive *Leptospira* spp. PCR outcome, Seoul hantavirus and *Rickettsia typhi* seropositive outcomes as dependent variables, had 1125, 1093 and 1114 complete observations respectively.

## Results

### Trapped rodents according to their species and trapping sites

**Figure 1:**
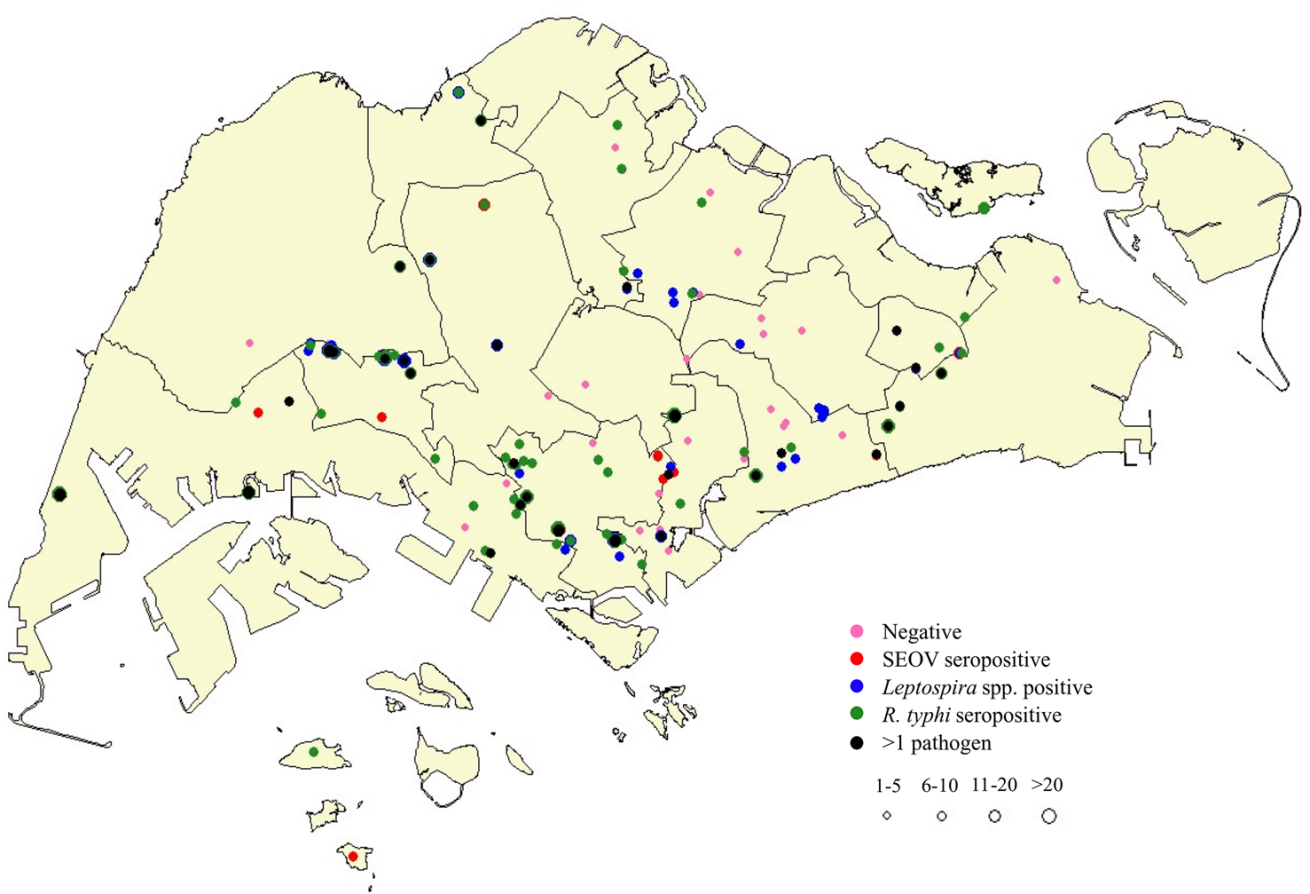
Spatial distribution of rodent trappings and prevalence of pathogens in *R. norvegicus* and *R. rattus* in Singapore, 2006–2008. Demarcations represent town councils.  : rodents with no infection;  : rodents seropositive for SEOV; : rodents positive for *Leptospira* spp.; : rodents seropositive for *R. typhi*; : rodents positive for more than one type of pathogen. The number of rodents trapped was reflected by the size of the circle. *Abbreviations*: SEOV, Seoul hantavirus. Map created using the Free and Open Source QGIS. QGIS.org, 2021. QGIS 2.18.25. Geographic Information System. QGIS Association. http://www.qgis.org.

Throughout Singapore, a total of 1143 rodents were trapped over a 2-year 2-month period. These rodents were trapped from 10 unique landscape structures, spanning over 14 town councils of the total 16 town councils in Singapore (Fig. 1). Species identification of 1143 rodents was done by sequencing, resulting in the identification of *Rattus norvegicus* (*n* = 990), *Rattus rattus* (*n* = 136), *Rattus tiomanicus* (*n* = 13), *Rattus exulans* (*n* = 3) and *Rattus tanezumi* (*n* = 1). Two species, *Rattus norvegicus* and *Rattus rattus*, were selected for further analysis, as other species were present in low numbers.

Rodents were trapped in the following landscape structures: Residential- public (*n* = 700), Residential- private (*n* = 25), Food establishments (*n* = 76), Construction sites (*n* = 44), Industrial areas (*n* = 47), Commercial sites (*n* = 118), Schools (*n* = 10), Army Camps (*n* = 46), Parks (*n* = 35), Shipyards (*n* = 25) (Table 1).

**Table 1:** Distribution of the captured *Rattus rattus* and *Rattus norvegicus* across the urban landscape and basic characteristics of the rodents.

Variable	*Rattus norvegicus*	*Rattus rattus*
**Landscape structure**		
Army camps	8	38
Commercial sites	117	1
Construction sites	24	20
Food establishments	76	0
Industrial areas	41	6
Parks	8	27
Residential- private	16	9
Residential- public	699	1
Schools	1	9
Shipyards	0	25
**Total number**	990	136
**Sex**		
Male	441 (44.6%)*	63 (46.3%)*
Female	530 (53.5%)*	71 (52.2%)*
**Mean body weight (g)**	187.93	112.37

*Some rodents have incomplete data; sex of rodents was not determined for 19 *Rattus norvegicus* and 2 *Rattus rattus*.

### Pathogenic and intermediate *Leptospira* spp.

The general prevalence of *Leptospira* spp. in tested rodents was 42.4%, with a higher prevalence observed in *R. norvegicus* (46.8%) compared with *R. rattus* (10.4%). The lowest prevalence of *Leptospira* spp. was found to be in schools (0.0%) and the highest prevalence to be in industrial areas (53.2%). In bivariate analyses, *Leptospira* spp. infection was associated with landscape structure, species, sex and weight of rodents (Table 2). Rodents captured in army camps (OR = 0.19, 95% CI: 0.08–0.40), construction sites (OR = 0.10, 95% CI: 0.03–0.26), food establishments (OR = 0.47, 95% CI: 0.28 to 0.78, parks (OR = 0.17, 95% CI: 0.06–0.41) and private residential developments (OR = 0.20, 95% CI: 0.06–0.52) showed a reduced prevalence of infection when compared with public residential developments (Table 2). *R. norvegicus* had 7.06 times (OR = 7.06, 95% CI: 4.20–12.7) higher likelihood than *R. rattus* to be infected, whereas female rodents were 1.38 times (OR = 1.38, 95% CI: 1.09–1.76) more likely than male rodents to be infected. The prevalence of *Leptospira* spp. infection increases for every 1 g increase in body weight for both *R. rattus* (OR = 1.03, 95% CI: 1.01–1.05) and *R. norvegicus* (OR = 1.009, 95% CI: 1.008–1.011) (Table 2).

**Table 2:** Bivariable logistic regression analysis for *Leptospira* spp. detection in *Rattus rattus* and *Rattus norvegicus*.

Explanatory variables	Odds Ratio	95% Conf. interval ^a^	*p*-value
**Landscape structure**			
Residential- public ^b^	Ref	–	–
Army camps	0.19	0.08, 0.40	** < 0.001**
Commercial sites	0.81	0.55, 1.20	0.3
Construction sites	0.10	0.03, 0.26	** < 0.001**
Food establishments	0.47	0.28, 0.78	**0.004**
Industrial areas	1.17	0.65, 2.13	0.6
Parks	0.17	0.06, 0.41	** < 0.001**
Residential- private	0.20	0.06, 0.52	**0.003**
Schools ^c^	–	–	–
Shipyards	0.81	0.35, 1.80	0.6
**Species**			
*Rattus rattus* ^b^	Ref	–	–
*Rattus norvegicus*	7.06	4.20, 12.7	** < 0.001**
**Sex**			
Male ^b^	Ref	–	–
Female	1.38	1.09, 1.76	**0.008**
**Weight (g)**			
*Rattus rattus*	1.03	1.01, 1.05	** < 0.001**
*Rattus norvegicus*	1.009	1.008, 1.011	** < 0.001**

Significant values (*P* < 0.05) are shown in bold.

^a ^95% Confidence Interval.

^b ^Reference category.

^c ^Omitted from the model because no *Leptospira* infection was detected at this site.

Multivariable logistic regression model for rodents with positive *Leptospira* spp. PCR outcome (Table 3) included the following three variables that were found to be statistically significant (*P* < 0.05) in the bivariate analysis: landscape structure, weight, and sex. Although the association between species of rodents and *Leptospira* spp. infection was statistically significant in the bivariate analysis, it was not included in the multivariate model due to collinearity (VIF > 2.0). Landscape structure, adjusted for weight and sex, was a statistically significant factor associated with *Leptospira* spp. infection in captured rodents. Prevalence of infection was increased in rodents captured in commercial sites (AOR = 2.91, 95% CI: 1.82–4.68), whereas rodents captured in army camps (AOR = 0.33, 95% CI: 0.12–0.76), construction sites (AOR = 0.19, 95% CI: 0.05–0.54) and parks (AOR = 0.18, 95% CI: 0.04–0.53) showed a reduced prevalence when compared with public residential developments (Table 3). *Leptospira* spp. infection was also independently associated with weight and sex of rodents. The prevalence of *Leptospira* spp. infection increases for every 1 g increase in body weight (AOR = 1.010, 95% CI: 1.009–1.012), whereas female rodents were approximately 1.5 times (AOR = 1.51, 95% CI: 1.14–2.01) more likely than male rodents to be infected (Table 3).

**Table 3:** Multivariable logistic regression model for *Leptospira* spp. detection in *Rattus rattus* and *Rattus norvegicus*.

Explanatory variables	Adjusted odds ratio	95% Conf. interval ^a^	*p*-value
**Landscape structure**			
Residential- public ^b^	Ref	–	–
Army camps	0.33	0.12, 0.76	**0.015**
Commercial sites	2.91	1.82, 4.68	** < 0.001**
Construction sites	0.19	0.05, 0.54	**0.005**
Food establishments	0.55	0.29, 1.00	0.053
Industrial areas	1.27	0.63, 2.29	0.5
Parks	0.18	0.04, 0.53	**0.006**
Residential- private	0.33	0.07, 1.10	0.33
Schools ^c^	–	–	–
Shipyards	1.13	0.47, 2.65	0.8
**Sex**			
Male ^b^	Ref	–	–
Female	1.51	1.14, 2.01	**0.004**
**Weight (g)**	1.010	1.009, 1.012	** < 0.001**

Significant values (*P* < 0.05) are shown in bold.

^a^ 95% Confidence Interval.

^b^ Reference category.

^c^ Omitted from the model because no *Leptospira* infection was detected at this site.

### Seoul hantavirus

The overall seroprevalence of Seoul hantavirus in tested rodents was 35.5%, with *R. norvegicus* (37.5%) having a higher seroprevalence than *R. rattus* (20.6%). SEOV seroprevalence ranged between 9.1% (construction sites)—46.8% (industrial areas) for the various landscape structures. In bivariate analyses, SEOV infection was associated with landscape structure, species, sex and weight of rodents (Table 4). Rodents captured in army camps (OR = 0.47, 95% CI: 0.22–0.93) and construction sites (OR = 0.16, 95% CI: 0.05–0.41) showed a reduced prevalence of infection when compared with public residential developments (Table 4). *R. norvegicus* had 2.34 times (OR = 2.34, 95% CI: 1.52–3.71) higher likelihood than *R. rattus* to be infected, whereas female rodents were 1.56 times (OR = 1.56, 95% CI: 1.21–2.01) more likely than male rodents to be infected. The prevalence of SEOV infection increases for every 1 g increase in body weight for both *R. rattus* (OR = 1.02, 95% CI: 1.01–1.03) and *R. norvegicus* (OR = 1.006, 95% CI: 1.005–1.07) (Table 4).

**Table 4:** Bivariable logistic regression analysis for Seoul hantavirus antibody detection in *Rattus rattus* and *Rattus norvegicus*.

Explanatory variables	Odds ratio	95% Conf. interval ^a^	*p*-value
**Landscape structure**			
Residential- public ^b^	Ref	–	–
Army camps	0.47	0.22, 0.93	**0.039**
Commercial sites	1.10	0.73, 1.65	0.6
Construction sites	0.16	0.05, 0.41	** < 0.001**
Food establishments	0.69	0.40, 1.15	0.2
Industrial areas	1.44	0.79, 2.61	0.2
Parks	0.55	0.23, 1.19	0.15
Residential- private	0.35	0.10, 0.93	0.056
Schools	0.18	0.01, 0.98	0.11
Shipyards	2.09	0.94, 4.77	0.073
**Species**			
*Rattus rattus* ^b^	Ref	–	–
*Rattus norvegicus*	2.34	1.52, 3.71	** < 0.001**
**Sex**			
Male ^b^	Ref	–	–
Female	1.56	1.21, 2.01	** < 0.001**
**Weight (g)**			
*Rattus rattus*	1.02	1.01, 1.03	** < 0.001**
*Rattus norvegicus*	1.006	1.005, 1.07	** < 0.001**

Significant values (*P* < 0.05) are shown in bold.

^a ^95% Confidence Interval.

^b ^Reference category.

Multivariable logistic regression model for SEOV antibody detection in rodents (Table 5) retained the following four variables that were shown to be statistically significant (*P* < 0.05) in the bivariate analysis: landscape structure, species, weight, and sex. Strong association was observed between landscape structure and SEOV infection in trapped rodents, with commercial sites (AOR = 2.77, 95% CI: 1.73–4.44) and shipyards (AOR = 6.61, 95% CI: 1.87–24.7) showing an increased prevalence of infection when compared with public residential developments (Table 5). *R. norvegicus* had 2.62 times (AOR = 2.62, 95% CI: 1.02–7.29) higher likelihood of SEOV infection compared with *R. rattus*, but at a borderline statistical significance (*P* = 0.054). SEOV infection was also independently associated with both the weight and sex of rodents. The prevalence of SEOV infection increases for every 1 g increase in body weight (AOR = 1.007, 95% CI: 1.006–1.009), whereas female rodents were 1.7 times (AOR = 1.70, 95% CI: 1.29–2.25) more likely than male rodents to be infected (Table 5).

**Table 5:** Multivariable logistic regression model for Seoul hantavirus antibody detection in *Rattus rattus* and *Rattus norvegicus*.

Explanatory variables	Adjusted odds ratio	95% Conf. interval ^a^	*p*-value
**Landscape structure**			
Residential- public ^b^	Ref	–	–
Army camps	1.82	0.63, 5.45	0.3
Commercial sites	2.77	1.73, 4.44	** < 0.001**
Construction sites	0.40	0.11, 1.12	0.11
Food establishments	0.80	0.44, 1.42	0.5
Industrial areas	1.75	0.89, 3.42	0.10
Parks	1.81	0.61, 5.38	0.3
Residential- private	0.64	0.14, 2.18	0.5
Schools	0.99	0.05, 7.08	> 0.9
Shipyards	6.61	1.87, 24.7	**0.004**
**Species**			
*Rattus rattus* ^b^	Ref	–	–
*Rattus norvegicus*	2.62	1.02, 7.29	**0.054** ^c^
**Sex**			
Male ^b^	Ref	–	–
Female	1.70	1.29, 2.25	** < 0.001**
**Weight (g)**	1.007	1.006, 1.009	** < 0.001**

Significant values (*P* < 0.05) are shown in bold.

^a ^95% Confidence Interval.

^b ^Reference category.

^c ^Borderline statistical significance.

### *Rickettsia typhi*

The overall seroprevalence of *Rickettsia typhi* in tested rodents was 32.2%, with *R. norvegicus* (32.6%) showing slightly higher seroprevalence than *R. rattus* (29.8%).

The lowest seroprevalence of *R. typhi* was found to be in construction sites (9.1%) and the highest in schools (60%). In bivariate analyses, *R. typhi* infection was associated with landscape structure and weight of rodents (Table 6). Rodents captured in commercial sites (OR = 0.28, 95% CI: 0.16–0.48) and construction sites (OR = 0.18, 95% CI: 0.05–0.45) showed a reduced prevalence of infection, whereas rodents trapped in industrial areas (OR = 2.05, 95% CI: 1.13–3.73) and shipyards (OR = 2.29, 95% CI: 1.03–5.24) displayed a higher prevalence of infection when compared with public residential developments (Table 6). The prevalence of *R. typhi* infection increases for every 1 g increase in body weight for both *R. rattus* (OR = 1.007, 95% CI: 1.001–1.013) and *R. norvegicus* (OR = 1.005, 95% CI: 1.004–1.006) (Table 6). In addition, species and sex of rodents did not influence prevalence of *R. typhi* infection.

**Table 6:** Bivariable logistic regression analysis for *Rickettsia typhi* antibody detection in *Rattus rattus* and *Rattus norvegicus*.

Explanatory variables	Odds ratio	95% Conf. interval ^a^	*p*-value
**Landscape structure**			
Residential- public ^b^	Ref	–	–
Army camps	0.90	0.46, 1.68	0.7
Commercial sites	0.28	0.16, 0.48	** < 0.001**
Construction sites	0.18	0.05, 0.45	**0.001**
Food establishments	0.66	0.38, 1.10	0.12
Industrial areas	2.05	1.13, 3.73	**0.018**
Parks	0.42	0.15, 0.96	0.056
Residential- private	0.50	0.16, 1.27	0.2
Schools	2.70	0.77, 10.7	0.13
Shipyards	2.29	1.03, 5.24	**0.043**
**Species**			
*Rattus rattus* ^b^	Ref	–	–
*Rattus norvegicus*	1.15	0.78, 1.73	0.5
**Sex**			
Male ^b^	Ref	–	–
Female	1.21	0.94, 1.56	0.14
**Weight (g)**			
*Rattus rattus*	1.007	1.001, 1.013	**0.035**
*Rattus norvegicus*	1.005	1.004, 1.006	** < 0.001**

Significant values (*P* < 0.05) are shown in bold.

^a ^95% Confidence Interval.

^b ^Reference category.

Multivariable logistic regression model for *R. typhi* antibody detection in rodents (Table 7) retained the following two variables that were shown to be statistically significant (*P* < 0.05) in the bivariate analysis: landscape structure and weight. Higher prevalence of *R. typhi* infection was observed in rodents captured in schools (AOR = 4.10, 95% CI: 1.14–16.4), industrial sites (AOR = 2.10, 95% CI: 1.13 to 3.9) and shipyards (AOR = 2.48, 95% CI: 1.08–5.75), whereas rodents trapped in commercial sites (AOR = 0.45, 95% CI: 0.24–0.77) and construction sites (AOR = 0.26, 95% CI: 0.08–0.68) presented a lower prevalence when compared to public residential developments (Table 7). The prevalence of *R. typhi* infection also increases for every 1 g increase in body weight (AOR = 1.004, 95% CI: 1.003–1.005). No association was found between *R. typhi* infection and either species or sex of rodents.

**Table 7:** Multivariable logistic regression model for *Rickettsia typhi* antibody detection in *Rattus rattus* and *Rattus norvegicus*.

Explanatory variables	Adjusted odds ratio	95% Conf. interval ^a^	*p*-value
**Landscape structure**			
Residential- public ^b^	Ref	–	–
Army camps	1.27	0.64, 2.41	0.5
Commercial sites	0.45	0.24, 0.77	**0.006**
Construction sites	0.26	0.08, 0.68	**0.013**
Food establishments	0.72	0.40, 1.23	0.2
Industrial areas	2.10	1.13, 3.90	**0.018**
Parks	0.63	0.23, 1.50	0.3
Residential- private	0.71	0.23, 1.84	0.5
Schools	4.10	1.14, 16.4	**0.033**
Shipyards	2.48	1.08, 5.75	**0.031**
**Weight (g)**	1.004	1.003, 1.005	** < 0.001**

Significant values (*P* < 0.05) are shown in bold.

^a ^95% Confidence Interval.

^b ^Reference category.

### *Yersinia pestis*

*Yersinia pestis* was not detected in the rodents tested.

## Discussion

*Rattus norvegicus* and *Rattus rattus* are the two commonly found rodent species in Singapore. *R. norvegicus* being the dominant species in Singapore, accounted for 86.6% of our trapped rodents, is also found in Southeast Asia and throughout the world^74–77^. Both species of rodents have adapted to occupy a wide variety of habitats where food and shelter may be found. Our study showed that most of the *R. norvegicus* were trapped in public residential developments (70.6%), while *R. rattus* were scattered across different urban habitats, with most of the *R. rattus* trapped in army camps (27.9%). *R. norvegicus* are good swimmers and their presence are often associated with sewer systems^12^, like those present in public residential developments. On the contrary, *R. rattus* are good climbers, often found in elevated settings such as ceiling and wall cracks of poorly maintained buildings and trees^12,18^, indicating that army camps may present the suitable environmental features favouring *R. rattus.* This confirmed the differential habitat preferences of *R. norvegicus* and *R. rattus*, with *R. norvegicus* preferring wet habitats, and *R. rattus* favouring drier habitats^15,19^.

This study revealed the presence of three rodent-borne pathogens: *Leptospira* spp., Seoul hantavirus and *Rickettsia typhi* in Singapore’s rodent population. Studies have shown that urban landscapes provide opportunities for increased rodent density^78,79^ and circulation of pathogens^80^. In addition, our study discovered that certain landscapes and environmental factors were associated with an increased probability of rodent infestation and occurrence of pathogens than others. Public residential developments in particular presented a concerning issue as shown by the large number of captured and infected rodents in the vicinity. This could pose a potential risk of transmitting diseases to humans, as these residential developments provided habitat and environmental features that encouraged rodents to feed and breed in close proximity to the human population.

Public residential development is one of the landscape structures with high population density, as more than 80% of Singapore’s population resides within these areas^81^. These high-rise buildings have a centralised rubbish chute system whereby rubbish is dropped into a large collection bin at the bottom of the refuse chute. Rodents could gain access to these bins through broken gully traps connected to the sewer system^82^. Accessible garbage through faulty gully traps has been identified as an environmental factor associated with rodent infestations^12^. Rodents congregating at these areas that provide shelter, water and food create opportunities for direct and indirect transmission of pathogens within the rodent population.

Chronically infected rodents are asymptomatic, and they can shed pathogenic *Leptospira* spp. and SEOV into the environment through their urine^3,83–86^. Outside a host, these pathogens thrive in the warm and moist surroundings, contaminating rodents’ living spaces including their food and water sources^87,88^. Transmission between rodents may also occur through direct contact with an infected rodent during social interactions such as grooming^84,89^ or fighting^3,74,90^, with the latter resulting in open wounds which can be infected by the contaminated environment. Garbage dumps and sewers are some of the habitats favoured by *R. norvegicus*, explaining the strong association between *R. norvegicus* and increased prevalence of *Leptospira* spp. and SEOV infections. These findings are in agreement with previous studies showing a high *Leptospira* spp. prevalence in *R. norvegicus* compared with *R. rattus*^91,92^.

Unlike SEOV and *Leptospira* spp., *R. typhi* is primarily transmitted by the Oriental rat flea (*Xenopsylla cheopis*). Fleas become infected when they feed on an infected rodent host^21^. As fleas usually feed and defecate at the same time, infected fleas feeding on other rodents and rubbing the infected flea faeces into the bite wounds or other wounds, could spread *R. typhi* to them^21^. Interestingly, our study revealed that 90% of the rodents caught in schools were *R. rattus*, and they had the highest likelihood of *R. typhi* infection, but not *Leptospira* spp. nor SEOV infection. On the other hand, 100% of the rodents caught in shipyards were also *R. rattus*, but they have been associated with an increased prevalence of both SEOV and *R. typhi* infections. Other than the presence of both fleas as vectors and rodents serving as hosts for *R. typhi* transmission, further investigation on the microenvironmental factors will be required to provide insight into environmental features conducive for transmission of *R. typhi*. It is also crucial to examine the habitats of *R. rattus* in shipyards to identify the factors promoting the circulation of rodent-borne pathogens.

Singapore reported that construction labourers accounted for 49% of the locally acquired human leptospirosis cases from 2012 to 2015 and a large proportion of the local murine typhus cases^53,93^. Construction workers are prone to sustain skin abrasions, which may increase the risk of infection. However, our study showed that the rodents trapped in construction sites had one of the lowest prevalence of *Leptospira* spp., SEOV and *R. typhi* infections. Therefore, both the occupational and non-occupational environments of the construction workers should be considered when determining the environmental factors associated with pathogen exposure risk. Housing types available for construction workers include temporary structures at construction sites, such as construction temporary quarters (CTQs), or a more long-term housing such as quick-build dormitories (QBDs) or purpose-built dormitories (PBDs)^94^. The temporary living quarters provided for the construction workers are often overcrowded, unhygienic and infested with rodents^53^. Rodent trapping and sampling at workers’ housing with suboptimal living conditions will be included in our future studies for better surveillance and interventions.

Previous studies have shown that male rodents had a higher chance of getting infected than female rodents as male rodents were more likely to engage in aggressive interactions than female rodents^88,95^. Other studies suggested that female rodents were more prone to infection, while some argued that both sexes were equally susceptible to possible infections^15,84^. In contrast, our study found higher prevalence of *Leptospira* spp. and SEOV infections in female rodents, while no difference in the prevalence of *R. typhi* infection was observed between the sexes. These conflicting research evidence may be hinting that the prevalence of infection could be more closely associated with habitat conducive for the transmission of pathogens, where both sexes are equally exposed to possible infections, than sex differences.

We reported that the increasing weight of rodents was strongly associated with prevalence of rodents being infected with any of the three rodent-borne pathogens. Our findings are consistent with previous reports stating that social and hierarchical interactions within the colony and exploratory behaviour of larger rodents^75^ could increase transmission events of these pathogens^15,76^. However, no clear association between weight of rodents and prevalence of *R. typhi* infection was observed in a previous study carried out in Baltimore, USA^96^. These discrepancies highlight that the risk factors had a varying extent of influence on prevalence of infection, and that it is important to consider the habitat and behaviour of rodents when determining the likelihood of infection.

When we compare to other urban rodent studies, the seroprevalence of *R. typhi* (32.2%) in tested rodents was similar to that in Jakarta, Indonesia (38.5%)^97^, but higher than that in Spain (21.2%)^98^. The seroprevalence of SEOV amongst the rodents was slightly higher (35.5%) than what was previously reported (26%) in a serosurvey conducted in Singapore in 1989^7^. *Leptospira* spp. was detected in over 40% of the rodents sampled, lower than that reported in Brazil^28^, but higher than that reported in Tokyo, Japan^99^. Despite having high prevalence of *Leptospira* spp., SEOV and *R. typhi* infections in Singapore’s rodent population, the reported annual incidences of the diseases in humans caused by these rodent-borne pathogens are relatively low. This could be attributed to the relative infrequent occurrence of rodents within residential homes compared to that on the streets which may reduce human-rodent contact and hence reduce the risk of acquiring the diseases^100^.

The main limitation of the study was sampling bias due to the use of opportunistic sampling strategy, which could lead to an over- or under-estimation of the pathogen prevalence. Moreover, opportunistic sampling reduced our ability to estimate rodent infestation rates at the different landscape structures, which would have strengthened our findings. The dominant pathogenic and/or intermediate *Leptospira* species circulating in Singapore’s rodent population was also not elucidated. This information is critical in understanding the epidemiology of leptospirosis which is the relationship between prevalent *Leptospira* species and the species of rodent reservoirs and their habitats. In addition, the prevalence of SEOV and *R. typhi* infections were determined by detecting pathogen-specific antibodies, and the respective pathogen genomes were not amplified. The current infection status of the rodents cannot be determined, as being seropositive for Seoul hantavirus and *R. typhi* could be due to previous infections. As a result, the pathogen load in our rodent samples cannot be determined. Rodent density and pathogen load are necessary to evaluate the degree of potential environmental contamination in an urban ecosystem, which would then influence the risk of human exposure, and the likelihood of transmitting rodent-borne diseases to humans within the shared environment. Further investigations are necessary to fully understand the role of rodents in disease transmission in their urban habitats and the impact on public health.

*Yersinia pestis* was not detected amongst the rodent population. Plague is not endemic to our nation and an article published in 1900 documented the introduction of plague to Singapore through trading port^101^. With strict preventive measures at our port, we prevented local transmission of *Yersinia pestis*. Nonetheless, Singapore remains vulnerable to the sporadic importation of *Yersinia pestis,* and constant vigilance is necessary.

## Conclusions

This study provides valuable insights on the presence of three rodent-borne pathogens in Singapore and their association with different landscape structures. *R. norvegicus* is the most dominant species in Singapore and is the most important reservoir for *Leptospira* spp. Rodents caught in public residential developments were the main interest as the habitat provided conducive conditions that contributed to an increased in rodent density and circulation of rodent-borne diseases in rodent population. Rodent infestation in close proximity to dense human settlements increases the risk of transmission of rodent-borne diseases to humans. Future studies focusing on the microenvironmental factors of individual urban landscape structures may reveal the determining factors associated with the circulation of pathogens in Singapore’s rodent population. Rodent management and sanitation strategies should be deployed to prevent further circulation of the pathogens among rodents and safeguard against transmission to human population.
